# Temperature and water availability induce chronic stress responses in zebra finches (*Taeniopygia guttata*)

**DOI:** 10.1242/jeb.247743

**Published:** 2024-11-20

**Authors:** Roger Colominas-Ciuró, Anna Kowalczewska, Małgorzata Jefimow, Michał S. Wojciechowski

**Affiliations:** ^1^ Department of Vertebrate Zoology and Ecology, Faculty of Biological and Veterinary Sciences, Nicolaus Copernicus University, 87-100 Toruń, Poland; ^2^ Department of Animal Physiology and Neurobiology, Faculty of Biological and Veterinary Sciences, Nicolaus Copernicus University, 87-100 Toruń, Poland

**Keywords:** Thermal stress, Water restriction, Leucocytes, H:L ratio, Zebra finch

## Abstract

Animals initiate physiological mechanisms to re-establish homeostasis following environmental stress. To understand how bird physiology responds to abiotic stress, we quantified changes in haematological markers of chronic stress response and body condition of male zebra finches (*Taeniopygia guttata*) acclimated for 18 weeks to hot and cool temperatures (daytime temperature: 40°C and 23°C) with water available *ad libitum* or restricted during half of the active phase. Ambient temperature induced greater chronic stress than restricted water availability. While cool compared with hot temperatures induced higher numbers of heterophils and heterophil to lymphocyte (H:L) ratios and reduced total leucocyte count, water restriction decreased the number of lymphocytes compared with water *ad libitum*. Body condition correlated with haematological parameters showing that birds with better condition had greater capacity to face environmental stress. Therefore, prolonged exposure to cool periods may result in chronic stress in zebra finches, especially if body condition is weakened.

## Teaser

**Summary:** Adverse ambient temperature and water availability trigger changes to leukocyte counts and stress response, which are not alleviated during prolonged acclimation in an arid-zone passerine.

## INTRODUCTION

Animals living in habitats with changing conditions can face environmental stressors such as unpredictable shifts in ambient temperature (*T*_a_), rainfall or the availability of resources that may invoke stress responses (Davis et al., 2008; Roast et al., 2019). Simultaneously, specific physiological and behavioural mechanisms are initiated to re-establish homeostasis (Kitaysky et al., 2007; Herring et al., 2011; Krams et al., 2011; Beaulieu et al., 2014; Wingfield et al., 2017). For instance, birds selectively exploited food resources leading to a better oxidative status and accumulated more energy reserves to cope with challenging conditions (e.g. *T*_a_ or unpredictable food availability) (Beaulieu et al., 2014; Laplante et al., 2019).

Changes in haematological markers [total leucocyte count and heterophil to lymphocyte (H:L) ratio] are a part of the complex animal response to environmental stressors such as thermal stress (Krams et al., 2011; Roushdy et al., 2020), restricted water availability (Chikumba et al., 2013; Brischoux et al., 2020; Navarrete et al., 2021), pathogens (Dunn et al., 2013) or social interactions (van der Meer and van Oers, 2015; Colominas-Ciuró et al., 2019). Total leucocyte count has been widely used to infer the health and condition as well as the chronic stress response of vertebrates in the wild (Davis et al., 2008; Davis and Maney, 2018). After a stressful situation (e.g. hot and cold spells, capture and handling, predatory attack), glucocorticoids such as corticosterone in birds increase within minutes, which is understood as the ‘signal’ for the leucocyte ‘downstream’ stress reaction (Davis and Maney, 2018). Hence, glucocorticoids trigger changes in leucocyte number (Davis et al., 2008) and, consequently, leucocyte reactions commence later and last longer (from >1 h to days, but it depends on the species) than changes in hormone concentrations (e.g. corticosterone; Gross and Siegel, 1983; Davis et al., 2008; Davis and Maney, 2018). For instance, exposure to thermal, hydric and handling stress led to a decrease of circulating leucocytes in captive birds (Parga et al., 2001; Scope et al., 2002; Davis, 2005; Chikumba et al., 2013; Roushdy et al., 2020; but see Krams et al., 2011, for thermal stress responses in the wild, although only heterophils and lymphocytes were assessed). The H:L ratio is also a reliable tool to assess chronic stress as leucocyte reactions redistribute lymphocytes from the blood to other body compartments and bring an influx of heterophils into the blood during the stress response (Davis et al., 2008, and references therein). Previous research associated higher chronic stress responses (high H:L ratios) with environmental changes (e.g. *T*_a_ or water availability; Krams et al., 2011; Chikumba et al., 2013; Davis and Maney, 2018; Roast et al., 2019; Roushdy et al., 2020).

Animal body condition is also influenced by environmental stress (e.g. heat stress; Krause et al., 2018; Müller et al., 2011). Reduced resource availability decreases body mass (Liang et al., 2015) and impairs growth rate in birds (Øyan and Anker-Nilssen, 1996). Thus, when animals face adverse conditions, energy demands for self-maintenance or other purposes may be traded off against other energetically costly functions (Norris and Evans, 2000; Van de Crommenacker et al., 2012). Previous studies showed negative relationships between H:L ratio and body condition and fat and energy stores (Gladbach et al., 2010; Müller et al., 2011; Włodarczyk et al., 2018), suggesting that H:L ratio may also be informative of the nutritionally based physiological stress (Jakubas et al., 2015).

This study complements the results described by Wojciechowski et al. (2021). They found that prolonged acclimation to high *T*_a_ resulted in a lowering of metabolic rate, leading to more efficient evaporative heat loss and more precise body temperature regulation. To understand birds' stress response to changing environmental conditions, we quantified the changes in haematological markers of the chronic stress response (changes in leucocyte counts and H:L ratio) and body condition of zebra finches (*Taeniopygia guttata*) exposed to temperatures below and above their thermoneutral zone (thermoneutral zone for this cohort ranged between ∼34.9 and 37.5°C; Wojciechowski et al., 2021). For 18 weeks, birds were exposed to hot (40°C during the day, 23°C during the night) or cool (23°C throughout the day and night) temperatures with water available *ad libitum* or restricted during half of their active phase. Zebra finches inhabit arid areas (Zann, 1996), generally coping with heat and water scarcity, but during cool ambient temperatures they increase energy metabolism as a result of the higher thermoregulatory costs of maintaining constant body temperature (Cooper et al., 2019; Wojciechowski et al., 2021). Therefore, we hypothesized that cooler rather than warmer temperatures induce a higher stress response. Despite being arid-zone birds, zebra finches can better accommodate heatwaves (high *T*_a_ and dry conditions) if drinking water is available (Cooper et al., 2019). Thus, we hypothesized that water restriction would amplify the effect of acclimation to *T*_a_ below thermoneutrality, while it would have a moderate effect at high *T*_a_. As elevated chronic stress responses are associated with low total leucocyte counts (as a result of low numbers of lymphocytes and high numbers of heterophils), high H:L ratios and low body condition (Øyan and Anker-Nilssen, 1996; Davis et al., 2008; Davis and Maney, 2018; Liang et al., 2015), we predicted that: (i) zebra finches acclimated to temperatures below thermoneutrality (23°C) would show a high stress response by increasing the number of heterophils and H:L ratio but decrease total leucocyte count and the number of lymphocytes compared with those acclimated at *T*_a_=40°C, temperatures close to their upper critical temperature (*T*_uc_=37.5°C; Wojciechowski et al., 2021); (ii) this disruption in haematological parameters and body condition would be intensified in dehydration stress, especially at 23°C; and (iii) a better body condition would lead to lower stress (low H:L ratio) and, consequently, a lower number of heterophils and a higher number of lymphocytes. The lack of predicted differences would indicate that birds had enough time to recover homeostasis during acclimation to the different thermal and water treatments.

## MATERIALS AND METHODS

### Experimental work

All procedures were approved by the Local Committee for Ethics in Animal Research in Bydgoszcz (permit numbers 9/2018 and 26/2018). The study was done in 2018 on 36 male (2 years old) zebra finches, *Taeniopygia guttata* (Vieillot 1817), that were hatched and raised in captivity at the Max-Planck Institute for Ornithology in Seewiesen, Germany. To start the experiment with similar conditions, all birds were transferred to the animal facilities at the Nicolaus Copernicus University in Toruń (Poland) ∼1.5 months prior to the experiment and were kept indoors under constant conditions at a *T*_a_ of 23±2°C and 12 h photoperiod (lights on at 08:00 h). Throughout the initial acclimation, birds were fed *ad libitum* (commercial mix for small exotic graminivores, Karma Mix, Mała Egzotyka, Bieruń Nowy, Poland) and every 2 days were supplemented with fresh greens, hard-boiled eggs, eggshells and vitamin and amino acid mixture (Biosupervit, Biofaktor, Skierniewice, Poland). Water access was unrestricted. After initial acclimation, individuals were randomly assigned to one of four groups. Each group was housed in a separate flight cage (1.2×1.2×1.8 m) under an unchanged light:dark cycle but with different temperature and water treatments for 18 weeks. The first group (*N*=9) was kept under hot temperatures (40±2°C) during the light phase of the day and cool (23±2°C) temperatures at night, and with water available *ad libitum.* The second group (*N*=9) was kept under the same thermal conditions but with water restricted for the half of the light phase of the day, from 11:00 h to 17:00 h. The third group (*N*=8) was kept at 23±2°C during both day and night hours, with water available *ad libitum*. The fourth (*N*=10) was kept under the same temperature but with water restricted for half of the light phase (11:00 h to 17:00 h). This design was dictated by the gregarious nature of zebra finches, the need to facilitate their flight, as well as the experimental facility constraints. As a result, our design did not include true replicates *sensu* Marshall (2024), but useful subsamples (Marshall, 2024).

On the day of sampling after 18 weeks of experimental acclimation, all individuals were captured, weighed to ±0.1 g with an electronic balance (SPU402, Ohaus) and tarsus length was measured to ±0.1 mm with a calliper (dialMax, Wiha, Germany). Immediately after, a blood sample was taken from a brachial vein using a heparinized capillary tube (75±0.5 mm; BRAND^TM^). One drop of blood was smeared on a microscope slide, air-dried and fixed with 96% methanol. We randomly captured all individuals in a group at once and proceeded with bird manipulations (∼5 min per individual) before capturing individuals from the next group, selected at random. Overall, group sampling lasted <1 h to avoid handling stress effects on leucocytes (Cïrule et al., 2012). Next, all slides were stained with 1:10 slow Giemsa (0.5 gr Azur Eosin Methylene Blue, E Merck AG, Darmstadt, Germany; 50 ml methanol and 50 ml glycerol) and phosphate-buffered saline pH 7.2 for 40 min.

### Leucocyte count

The total leucocyte count for each blood smear was determined by counting all leucocytes (heterophils, eosinophils, monocytes, basophils and lymphocytes) found in 10 non-overlapping microscope fields under 400× magnification (Menéndez-Blázquez et al., 2021). In addition, leucocytes were counted by examining blood smears under 1000× magnification using an oil immersion objective with an Olympus CX21 microscope, and the proportion of the different leucocyte types was obtained by examining a total of 100 leucocytes per slide. In the present study, eosinophils (mean±s.d. 3.222±4.350), monocytes (2.667±2.777) and basophils (2.472±2.348) were not considered (frequency of occurrence <5%). All leucocyte counts in both methods (total leucocyte count under 400× magnification and under 1000× magnification) were done in a part of the smear where cells had been separated in a monolayer crossing the sample from bottom to top or from left to right to minimize differences in blood smear thickness. Finally, the ratio between heterophils and lymphocytes was calculated for all samples. All smears were examined by the same person (R.C.-C.).

### Statistical analyses

Total leucocyte count under 400× magnification and H:L ratio were log(*x*+1) transformed to meet the assumption of normal distribution of residuals of the linear models (LMs; Shapiro–Wilk tests, *P*≥0.169). Body condition was quantified as residuals from an ordinary least squares linear regression of body mass against tarsus length (Schulte-Hostedde et al., 2005). To analyse total leucocyte count under 400× magnification, H:L ratio and heterophil and lymphocyte count, four separate LMs were fitted with temperature and water availability as well as their interaction as explanatory variables and body condition as a covariate. Another LM was used to analyse body condition as a response variable fitted with acclimation temperature and water availability as well as their interaction as explanatory variables. Statistical significance was accepted at α<0.05. We did all statistical analyses and prepared graphs using R version 3.5.1 (http://www.R-project.org/) using packages ‘stats’, ‘car’ and ‘ggplot2’ (Fox and Weisberg, 2011; Wickham, 2016).

## RESULTS AND DISCUSSION

**Fig. 1.**
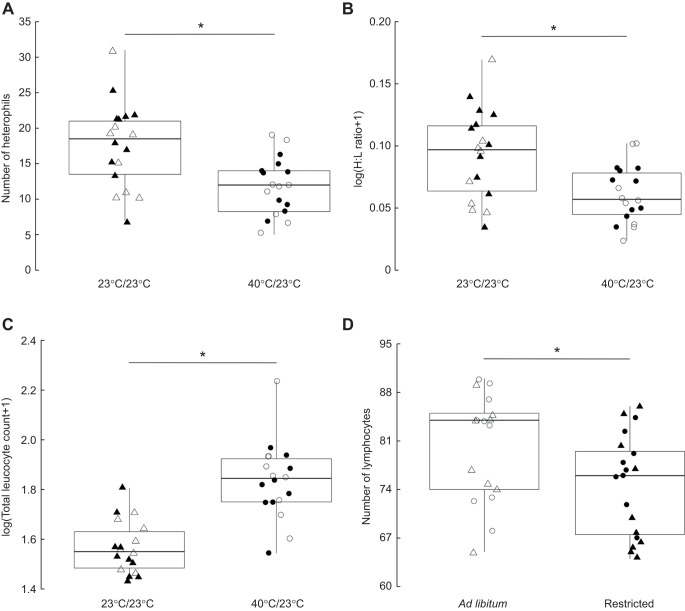
**Markers of the haematological chronic stress response in zebra finches (*Taeniopygia guttata*) acclimated to different ambient temperatures and water regimes.** (A) Heterophil count, (B) heterophil to lymphocyte (H:L) ratio, (C) total leucocyte count and (D) lymphocyte count of zebra finches acclimated to different ambient temperatures (cool, 23°C; and hot, 40°C) and different water treatments (*ad libitum* and restricted during half of the active phase). The top of each box indicates the third quartile (Q3), the horizontal thick line indicates the median, the bottom of the box indicates the first quartile (Q1) and whiskers indicate the maximum/minimum values (*P<0.05, linear models, LMs). Open circles refer to 40°C day/23°C night and *ad libitum* water (*n*=9); filled circles refer to 40°C/23°C and restricted water (*n*=9); open triangles refer to 23°C and *ad libitum* water (*n*=8); and filled triangles refer to 23°C and restricted water (*n*=10).

In the current study, we investigated haematological markers of the chronic stress response (changes in leucocyte count and H:L ratio) and body condition of zebra finches facing hot (40°C during the daytime and 23°C during the night) and cool (23°C throughout both day and night) temperatures with water available *ad libitum* or restricted during half of the active phase. Overall, our results suggest that low *T*_a_ is more stressful than water restriction. Acclimation to cool compared with hot temperatures induced a higher chronic stress response by increasing the number of heterophils and the H:L ratio, and decreasing the total leucocyte count regardless of water availability (Fig. 1A–C, Table 1; Table S1). These results concur with those of whole-body respirometry for this cohort, which showed that acclimation to 40°C during the daytime resulted in the lowering of metabolic rate (Wojciechowski et al., 2021). Regardless of temperature treatment, water restriction caused a stress response, manifested by a decrease in the number of lymphocytes (Fig. 1D, Table 1).

**Table 1.**
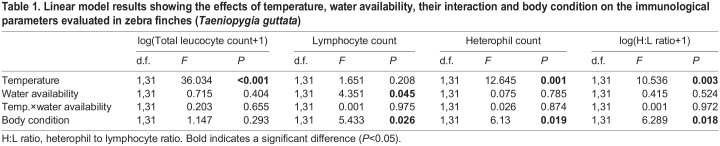
Linear model results showing the effects of temperature, water availability, their interaction and body condition on the immunological parameters evaluated in zebra finches (*Taeniopygia guttata*)

Haematological parameters have been widely used to study stress in birds because they are sensitive to environmental stressors (Davis et al., 2008; Krams et al., 2011; Chikumba et al., 2013; Dunn et al., 2013; van der Meer and van Oers, 2015; Colominas-Ciuró et al., 2019). In birds, chronic stress, such as changes in *T*_a_ or water regimes, is reflected by higher numbers of heterophils, lower numbers of lymphocytes and, consequently, higher H:L ratios (e.g. Davis et al., 2008; Davis and Maney, 2018; Krams et al., 2011; Cīrule et al., 2012; Colominas-Ciuró et al., 2019), or a general decrease of circulating leucocytes (Parga et al., 2001; Scope et al., 2002; Davis, 2005; Chikumba et al., 2013; Roushdy et al., 2020). In our study, zebra finches kept at 23°C throughout the day experienced greater chronic stress than those acclimated to hotter *T*_a_. The thermoneutral zone for this cohort ranged between ∼34.9 and 37.5°C (Wojciechowski et al., 2021), and their energy demands are expected to be higher at cooler temperatures because of the energetic costs of thermoregulation (Calder, 1964; Cooper et al., 2019; Wojciechowski et al., 2021). It is therefore plausible that a constant *T*_a_ of 23°C induced a chronic stress response by, first, increasing corticosterone concentration, which would have triggered a long-lasting haematological disruption (Davis et al., 2008; Davis and Maney, 2018), as shown by an increased number of heterophils and H:L ratio, and a decreased total leucocyte count in birds acclimated to temperatures below thermoneutrality (Fig. 1A–C). These results corroborate with the absence of corticosterone differences in zebra finches kept at different hot temperatures (i.e. mean maximum *T*_a_ of 42.7°C and 31.4°C: Cooper et al., 2020; and maximum *T*_a_ of 43.18±0.12°C and 34.42±0.12°C: Xie et al., 2017), probably because these *T*_a_ were all around thermoneutrality. These results show that zebra finches successfully cope with hot spells (lower stress levels at hot temperatures, H:L ratios ∼0.155±0.06; Table S1) in contrast to other Australian desert birds (doves and budgerigars), which raised the same marker of stress (H:L ratios from 1.37±0.92 to 4.46±2.71) after exposure to similar hot *T*_a_ (Xie et al., 2017). At high *T*_a_, doves and budgerigars engage effective mechanisms of gular flutter or cutaneous evaporative water loss, while zebra finches instead rely on respiratory water loss. These two strategies may involve different energetic costs (McKechnie et al., 2021), which could reflect H:L ratio differences as higher metabolic rates are associated with higher H:L ratios (Colominas Ciuró et al., 2019). Thus, prolonged acclimation to cool *T*_a_ way below thermoneutrality (23°C) caused homeostatic disruption (higher stress) in zebra finches, showing no recovery during the study period. Under a scenario of climate change, zebra finch resilience and potential adaptation to hot conditions might benefit them in the face of the impacts of global warming. Finally, this result is also notable for animal experimentation, as *T*_a_ values of ∼23°C are frequently used as housing temperature for such birds in captivity. Research on optimum housing conditions for this species to avoid stress is therefore necessary. Otherwise, homeostatic disruption might result in deleterious consequences for birds and biased scientific results.

We also predicted significant disruption of chronic stress haematological markers in water-restricted birds as dehydration stress reduces leucocyte count and increases H:L ratio (Zulkifli, 1999; Chikumba et al., 2013). However, our results indicate that restriction of water at least during half of the active phase triggers only changes in the number of lymphocytes (Fig. 1D). Environmental stressors depress the number of lymphocytes in peripheral circulating blood and induce an influx of heterophils from bone marrow (Johnstone et al., 2012). Also, the strength of the stress response is proportional to the concentration of glucocorticoids and H:L ratio (Van Dijk et al., 1979; Davis et al., 2008). Therefore, water restriction seems a milder stressor than ambient temperature because lymphocytes were the only disrupted leucocyte type. It is worth mentioning that water restriction occurs often in the wild and, thus, zebra finches could be naturally resilient by retreating into the shade, obtaining water from food or reducing their activity during the hottest periods of time (Wolf, 2000). Alternatively, dehydration may require more time to become a major stressor or allostasis may be taking place and some leucocytes are achieving stability. Overall, the usefulness of haematological parameter profiles as a reliable proxy of the chronic stress response under changing conditions seems to depend on both the specific new condition and the immunological plasticity with which birds respond.

**Fig. 2.**
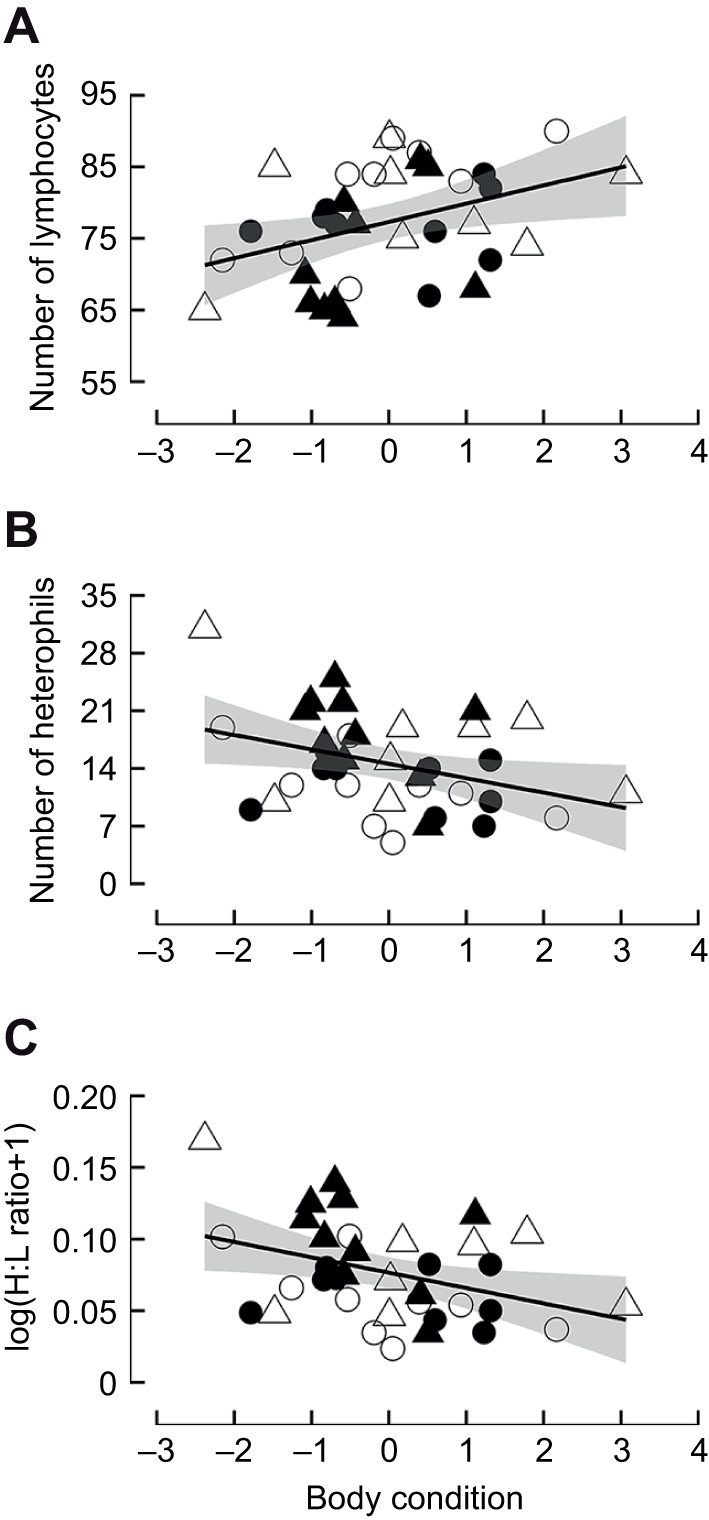
**Relationship of markers of the haematological chronic stress response to body condition in zebra finches acclimated to different ambient temperatures and water regimes.** (A) Lymphocyte count, (B) heterophil count and (C) H:L ratio of zebra finches acclimated to different ambient temperatures (cool, 23°C; and hot, 40°C) and different water treatments (*ad libitum* and restricted during half of the active phase). Because there was no effect of acclimation on the analysed relationships (LMs), data for all groups were pooled, but are graphed with different symbols: open circles refer to 40°C day/23°C night and *ad libitum* water (*n*=9); filled circles refer to 40°C/23°C and restricted water (*n*=9); open triangles refer to 23°C and *ad libitum* water (*n*=8); and filled triangles refer to 23°C and restricted water (*n*=10).

Body condition and mass influence physiology (e.g. H:L ratio; Gladbach et al., 2010) and behaviour (e.g. dominance rank, behaviour of migration; Duijns et al., 2017; Francis et al., 2018), and have been associated with environmental features (e.g. snowstorms; Krause et al., 2018). Although we found no significant differences in body condition under different treatments (temperature: *F*_1,32_<0.001, *P*=0.993; water availability: *F*_1,32_=0.221, *P*=0.642; temperature×water availability: *F*_1,32_=1.002, *P*=0.324), our results showed that body condition was negatively related to the number of heterophils and H:L ratio, and positively related to the number of lymphocytes (Fig. 2 and Table 1). Also, no association between body condition and total leucocyte count was found (Table 1). The immune system has different arms that often require different energetic demands (Lee and Klasing, 2004). On the one hand, baseline (or constitutive/innate) responses require high energy investment, involving fever and inflammation, as for heterophils, which are the first line of immune defence recognizing and eliminating foreign invaders by primitive and non-specific systems (Lee and Klasing, 2004; Buehler, 2005). On the other hand, the costs of adaptive immune responses are thought to be small, as for lymphocytes, which specifically recognize and recall antigens of pathogens (Lee and Klasing, 2004; Buehler, 2005). Our results show that individuals with better body condition present lower stress levels (H:L ratio) by investing more in the adaptive immune response (higher lymphocyte numbers), which is less energetically costly, and less in innate immunity (low heterophil numbers), which is comparatively more energetically expensive. A lower investment in producing heterophils might indicate a low perception (or burden) of parasites and pathogens in the new experimental ecosystem compared with the wild, which could be a result of captivity. Furthermore, the H:L ratio has been found to be informative of the nutritionally based physiological stress because it was negatively related to body condition, fat content and plasma metabolites (Gladbach et al., 2010; Müller et al., 2011; Włodarczyk et al., 2018). Our results therefore agree with previous studies because individuals in better condition showed lower stress levels (H:L ratio) and probably cope better with adverse environmental conditions.

In conclusion, acclimation to prolonged periods of cool *T*_a_ causes greater chronic stress than hot *T*_a_ and restricted water availability in small passerines inhabiting arid areas, showing no recovery during the study period. This reveals the physiological plasticity of such species to successfully overcome hot spells, which is essential to understand the impacts of climate change on biodiversity. However, individuals with better body condition also have a greater capacity to react and acclimate to environmental stress, suggesting that prolonged exposure to challenging conditions (cool *T*_a_ for zebra finches) may result in deleterious effects for animal populations, especially if body condition is weakened. Our results also call for a revision of housing conditions when zebra finches are kept at *T*_a_ around 20–25°C. It might be advantageous for birds to increase the ambient temperature in their facilities.

## Supplementary Material

10.1242/jexbio.247743_sup1 Supplementary information
