# Supplementary material for: Temperature and water availability induce chronic stress responses in zebra finches (Taeniopygia guttata)
Source: J Exp Biol. 2024 Nov 20;227(22):jeb247743. doi: 10.1242/jeb.247743 (PMC11607686; doi:10.1242/jeb.247743)
Supplement: Supplementary information [file jexbio-227-247743-s1.pdf]

Table S1. Descriptive statistics for all immunological parameters evaluated (total leucocyte counts, the number of lymphocytes, the number of heterophils, heterophil to lymphocyte (H : L) ratio) in zebra finches (*Taeniopygia guttata*) sorted by experimental treatments (40-23°C and *ad libitum* H_2O; 40-23°C and restricted H_2O; 23°C and *ad libitum* H_2O; and 23°C and restricted H_2O).

	40-23°C, H_2O *ad libitum* (N = 9)		40 -23°C, H_2O restricted (N = 9)		23°C, H_2O *ad libitum* (N = 8)		23°C, H_2O restricted (N = 10)	
	Mean ± SD	Min - Max	Mean ± SD	Min-Max	Mean ± SD	Min-Max	Mean ± SD	Min-Max
Total leucocyte counts	78.11 ± 38.25	39 - 171	65.67 ± 17.61	34 - 92	38 ± 8.07	28 - 50	36.10 ± 11.72	26 - 63
Lymphocytes	81.11 ± 8.04	68 - 90	76.78 ± 5.07	67 - 84	79.12 ± 7.81	65 - 89	72.7 ± 8.53	64 - 86
Heterophils	11.56 ± 4.67	5-19	11.89 ± 3.37	7 - 16	16.88 ± 7.08	10 – 31	18.10 ± 5.32	7 – 25
H : L ratio	0.15 ± 0.07	0.06 - 0.26	0.16 ± 0.05	0.08 - 0.21	0.22 ± 0.12	0.11 – 0.48	0.26 ± 0.09	0.08 – 0.38

Note: *SD* - standard error, *Min-Max* - minimum and maximum values.
